# Commentary: Relation Between Blood Pressure and Pulse Wave Velocity for Human Arteries

**DOI:** 10.3389/fphys.2019.01179

**Published:** 2019-09-12

**Authors:** Mohammad Yavarimanesh, Anand Chandrasekhar, Jin-Oh Hahn, Ramakrishna Mukkamala

**Affiliations:** ^1^Department of Electrical and Computer Engineering, Michigan State University, East Lansing, MI, United States; ^2^Department of Mechanical Engineering, University of Maryland, College Park, MD, United States

**Keywords:** blood pressure, pulse wave velocity (PWV), hemodynamics, arterial stiffness, cuff-less blood pressure measurement

Ma et al. recently proposed to relate blood pressure (P) and pulse wave velocity (PWV) for human arteries via a two-parameter, quadratic formula: *P* = α*PWV*^2^+β. Once α and β are determined, this formula could potentially be applied for cuff-less P measurement.

The authors (i) applied force balance to a thick-walled cylindrical tube under internal pressure and composed of elastic material characterized by the arterial strain-energy function of Fung to derive a relation between arterial cross-sectional area (A) and P; (ii) substituted this A-P relation into the Bramwell-Hill equation ($PWV=\sqrt{\left(A/\rho \right)\left(dP/dA\right)}$, where ρ is blood density) to yield a complicated relation between PWV and P; and (iii) made approximations to arrive at the reduced formula. They imply that the PWV can be measured anywhere in the arteries (see Figures 1, 5C,D), but several aspects of their derivation appear to assume the aorta. For example, the resulting PWV in Figure 4 represents aortic values [see Figure 3 of Reference values for arterial stiffness' collaboration, 2010 and Figure 19.15 of Vlachopoulos et al., 2011].

PWV in large arteries, which are relatively sparse in smooth muscle (Burton, 1954), may best track P. However, the Fung strain-energy function cannot predict the sigmoid-shaped A-P relation of the aorta (see p. 41 of Holzapfel et al., 2000), which is most prominent in youth (Hallock and Benson, 1937) and at *in situ* length (Bergel, 1960). Equation (10) in the authors' paper does not have positive and negative second derivatives for physical parameters, and, as shown in Figure 1A, cannot fit renowned human aortic A-P data (Hallock and Benson, 1937) well. Holzapfel et al. proposed a histologically-based strain-energy function with more parameters to produce sigmoid-shaped A-P relations (see Figure 16a of Holzapfel et al., 2000). King proposed a physical model based on force balance in a thin-walled cylindrical tube with elastomeric wall, which could also predict sigmoid-shaped A-P relations (see Figure 2 of King, 1946). Langewouters et al. reported that a simple arctangent fits human aortic A-P data particularly well, as shown in Figure 1B (Langewouters et al., 1984). Substituting this empirical function into the Bramwell-Hill equation yields the P-PWV formula:

(A) $$\begin{array}{l} PWV=0.357\sqrt{\pi P_{1}\left(1+{\left(\frac{P-P_{0}}{P_{1}}\right)}^{2}\right)\left(\frac{1}{2}+\frac{1}{\pi }\mathrm{atan}\left(\frac{P-P_{0}}{P_{1}}\right)\right)},\left(\text{A}\right) \end{array}$$

where PWV is in m/s and *P*_0_ and *P*_1_ are unknown parameters in mmHg. While the quadratic formula predicts concave-up curves, as shown in Figure 1C, Equation (A) here predicts concave-down curves whose shape changes with aging and approaches a line at higher P and in the elderly, as shown in Figure 1D. This two-parameter formula yields predictions similar to the corresponding formula of King (see Figure 3 of King, 1947) while affording a more convenient form.

**Figure 1:**
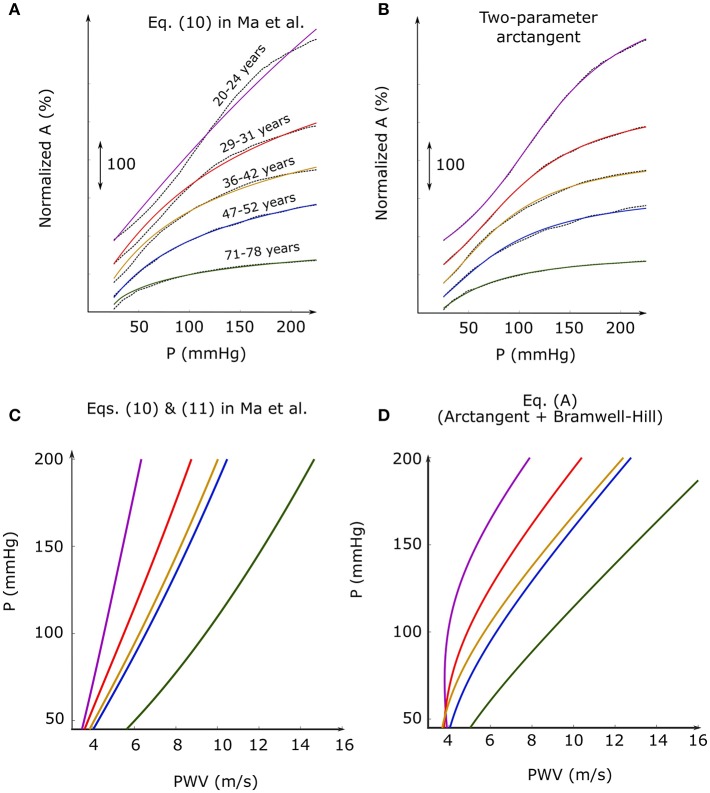
**(A,B)** Least squares fits of Equation (10) in the article of Ma et al. and two-parameter arctangent (solid-color) to human aortic cross-sectional area (A)-blood pressure (P) data (dash-black) for different age groups (Hallock and Benson, 1937). **(C,D)** Corresponding P-pulse wave velocity relations resulting from substituting the above model A-P relations into the Bramwell-Hill equation ($PWV=\sqrt{\left(A/\rho \right)\left(dP/dA\right)}$, where ρ is blood density).

In the practical case, a line relation may suffice, as the P range may be limited and confounding factors such as smooth muscle contraction and pre-ejection period may have a linearizing effect. Alternatively, these non-trivial factors, which are ignored by all of the formulas, could alter the non-linear form of the relation. It may therefore make most sense to identify both the form and parameters based on measured P-PWV pairs.

In sum, evidence suggests that, in the ideal case, the P-PWV relation for at least the relevant large arteries is given by the two-parameter formula of Equation (A) rather than a quadratic.

## Author Contributions

MY, AC, and RM prepared the paper. J-OH edited the paper.

### Conflict of Interest Statement

The authors declare that the research was conducted in the absence of any commercial or financial relationships that could be construed as a potential conflict of interest.
